# Boundary Spanning Behavior of Clinician-Teachers in the Classroom. An Observation Study

**DOI:** 10.5334/pme.1751

**Published:** 2025-08-06

**Authors:** Hiske Joanna Brouwer, Elco Buurma, Esther de Groot, Monika Louws, Manon Kluijtmans, Roger Anna Maria Joseph Damoiseaux, Margot Barry

**Affiliations:** 1 Department of General Practice & Nursing Science, Julius Center for Health Sciences and Primary Care, University Medical Center Utrecht, Utrecht University, Utrecht, The Netherlands; 2 Department of Education, University of Utrecht, Utrecht, The Netherlands; 3 Education Center, University Medical Center Utrecht, Utrecht University, Utrecht, The Netherlands

## Abstract

**Introduction:**

Clinician-teachers are engaged in both clinical practice and education. They positively influence student learning by connecting clinical practice and education. Most research into clinician-teacher’s dual role was performed in the clinical settings where practice and teaching are intertwined. The benefits of clinician-teachers’ dual role in the classroom-setting have been underexplored, whilst a large part of medical education is classroom-based. Using boundary work theory as a lens, this study aimed to illuminate clinician-teachers’ observable boundary spanning behavior integrating the clinical practice and medical education in the classroom.

**Methods:**

A qualitative observation study of classroom-teaching within postgraduate general practitioner specialty training at three Dutch medical institutes was conducted. Video recordings and transcripts of classroom teaching were analyzed using a structured observation schedule. Boundary spanning behavior was categorized into: boundary bridging, boundary making and boundary maintenance. Distinctions were made between verbal- and non-verbal behavior.

**Results:**

All three categories of boundary spanning behavior were observed. Clinician-teachers demonstrated boundary bridging by integrating their own clinical experiences, by normalizing students’ reported clinical experiences, by encouraging students’ sharing of clinical experiences and by encouraging students to apply theory in practice. Clinician-teachers demonstrated boundary making by accentuating discontinuities between clinical practice and educational information, and boundary maintenance by allowing transient differences to exist between the two settings for didactic reasons.

**Discussion:**

This observational study contributes to an understanding of how clinician-teachers use their experience as a clinician in classroom-teaching. These insights may contribute to faculty development fostering boundary-spanning teaching practices.

## Introduction

A clinician-teacher (CT) is a professional who actively works as both a clinician and teacher, applies theory to educational work, engages in education scholarship, and provides consultation to colleague health professionals on education issues [1]. In medical education, it is assumed that CT’s add value to students by connecting education and clinical practice [2]. In the clinical setting this is achieved through the CTs role-model function and their clinical visibility (the white coat) alongside their educational tasks. Students view CTs in the clinical setting as credible content experts [3,4,5,6]. In the classroom setting, the CTs credibility as content expert and their ability to connect education and clinical practice are less overtly visible, yet, also known to enhance student learning [7]. In medical education, few publications are concerned with the classroom setting, whilst a large part of medical education takes place within classrooms [8]. Available research relies on self-report data and shows that CTs connect theoretical lesson content with practice by giving clinical examples [9,10], and by sharing personal clinical experiences as well as the impact these had on them [11]. By doing this they are said to influence student learning [9,10,11] and also professional identity formation [12]. Research in the professional field of nursing describes how CTs connect the classroom and clinical settings by stimulating students’ reflection on clinical situations in the classroom, by concept mapping of issues arising in clinical practice and by journaling [13]. How CTs in medical education enact boundary spanning behavior beyond sharing their experiences is currently less clear in literature. Given the positive effects of CTs’ boundary spanning behavior on student learning in classroom settings, it is important to gain insight into how CTs’ boundary spanning behavior manifests. This may contribute to CTs conscious use of boundary spanning behavior and faculty development fostering this behavior.

The current insights on how CTs connect education and clinical practice arise from self-report data, which has inherent limitations, such as interviewees limited in-depth understanding of- or willingness to report on relevant phenomena [14]. It is also possible that experts act intuitively and are unconsciously competent, thereby not reporting on professional actions, which they consider as negligible, whilst these are relevant to the research. Observations might add to existing interview-based insights.

We operationalized the observation of boundary spanning behavior by drawing on ‘boundary work’ literature. This literature is multi-disciplinary including social- [15], organizational- [16,17,18,19], educational- [20], medical- [21] and legal [22] sciences, and refers to social processes through which individuals or groups interact with boundaries that demarcate social categories, identities or knowledge domains. Commonalities and differences in conceptualization of boundary spanning exist between these disciplines, whilst they all agree on the demarcations and boundaries that exist between contexts such as education and (clinical) practice. Boundary work literature offers insights into how these boundaries can be approached. Of the three approaches described, boundary bridging is most widely known and concerns itself with blurring the boundary between two settings. Boundary making concerns itself with the accentuation of existing discontinuities between settings. Boundary maintenance describes the creation of temporary boundaries with the purpose of achieving an educational result. In the next paragraphs, we elaborate on these three types of boundary spanning in medical education.

*Boundary bridging* involves activities in which the CT actively blurs sociocultural differences across settings and seeks connections to collaborate between differentiated expertise and responsibilities [15,16,20,22]. CT’s sharing their own clinical expertise, for example, could enlarge the learning potential for students, by providing students with insight into the culture, traditions and practices of the professional community in clinical practice [23]. CTs might be viewed as role models by being seen as an expert representative of the medical profession [23].

*Boundary making* involves activities in which the CT distinguishes two contexts from each other by defending boundaries from a self-oriented perspective [15,16,20,22]. For example, highlighting their inability to teach students in a classroom to manage emotions and stress in a critical medical situation. A CT emphasizing boundaries may be experienced by students as unpleasant or difficult, however reflection on these boundaries does create learning potential in a sense that students might be better prepared for the practice reality [24].

*Boundary maintenance* involves activities that create a purposeful temporary focus on differences between the two settings, for pedagogical or didactic reasons. For example, classroom teaching of a complex clinical procedure might focus on the mastery of individual parts of the procedure initially, whilst these are never executed separately in clinical practice. The differences between both settings are not presented as fixed (as is the case with boundary making), rather, an ‘elastic balance’ between differences is maintained through which some activities are at least temporarily separate [16,22].

### Research aim and question

The aim of this study is to observe whether and how CTs enact boundary spanning behavior in the classroom. The study is conducted in classroom education of post graduate general practitioner specialty training, using the above described categories of boundary spanning behaviors as a lens. The research question guiding this study was: *“How do clinician teachers enact boundary spanning behavior in a postgraduate general practitioner classroom setting?*

## Methods

### Study design, context and participants

We conducted a qualitative observation-based research of classroom teaching by CTs in the postgraduate specialty training for general practitioners (GPs) in the Netherlands. In this three-year program, GP specialty trainees receive weekly full-day classroom education alongside their four-day practice placement within a GP practice. The classroom education is facilitated by CTs, who actively work as GPs for at least one day a week and as teacher for at least two days a week. CTs receive didactical training for their teaching role. CT’s always co-teach together with a behavioral scientist or psychologist. As duos they teach the same small groups of approximately twelve GP trainees for a full year. All eight Dutch GP training institutes have comparable program structures and qualification requirements for CTs. We approached three GP training schools due to their accessibility to the researchers involved: UMC Utrecht, Radboud UMC Nijmegen and UMC Groningen. Eligible participants for our purposive sample were CTs with at least two years’ working experience as a CT. We aimed to observe well-established CT behavior. New CTs might possibly still becoming acquainted with the teacher-role and less able to span boundaries. Ethical approval was granted on by the ERB-NVMO on 27-08-2020 (NERB dossier number 2020.5.4). All CTs of the three institutes were invited to participate by e-mail. We met the aim of including six CTs, two from each training institute, and observed at least three lessons per CT.

### Data collection

**Figure 1:**
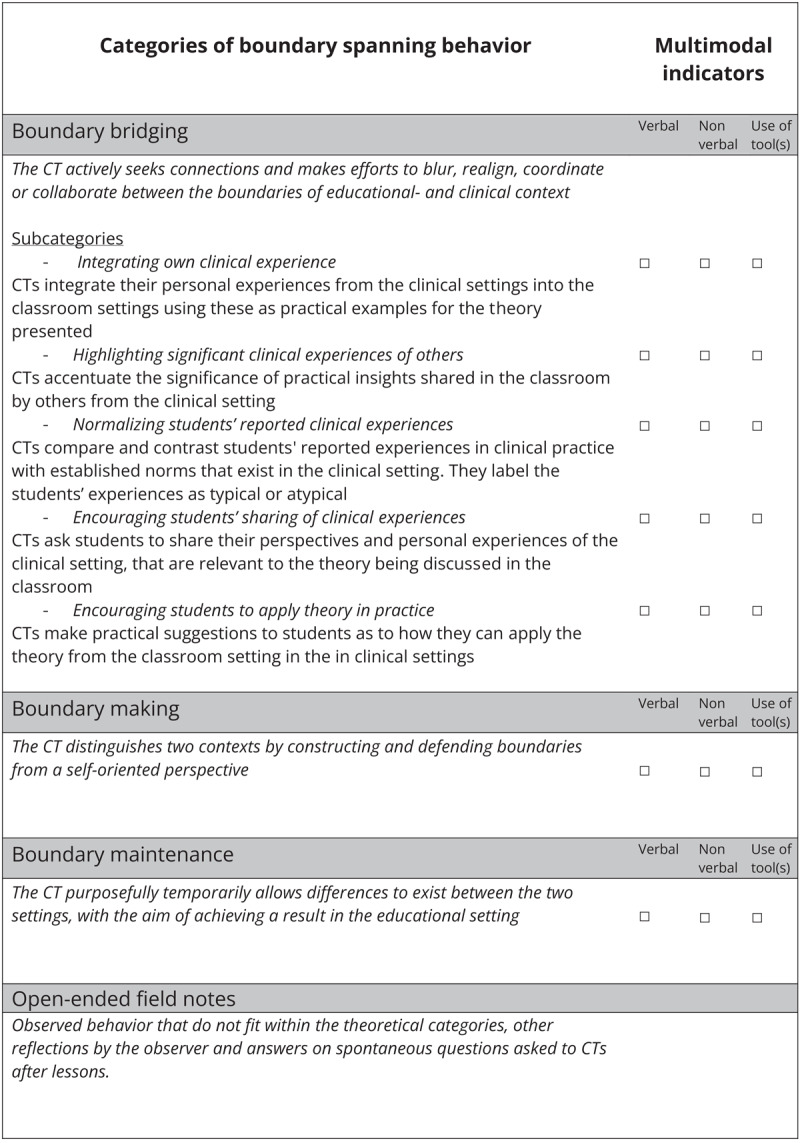
Final observation schedule for documentation and analysis of the boundary spanning behavior of CTs. CT: clinician-teacher.

Classroom observations were conducted between December 2021 and December 2023. During this time a government-imposed COVID-19 lockdown prolonged the anticipated data collection period and some observations were conducted during online lessons. The researcher HB was physically present as a non-participant observer for 18 of 22 classroom-based lessons and virtually present via Microsoft Teams for six online lessons. During online lessons HB could be seen on the screen throughout the lesson. Lessons were video and audio recorded. All persons present gave written informed consent prior to the recording and were given the possibility to withdraw their consent. This was the case for physical and online lessons. Withdrawal would result in (partial) deletion of their words from the transcripts of the audio- and video recordings. No one withdrew their consent. An observational instrument (see Figure 1) was developed based on the predefined theoretical framework of boundary spanning behavior described earlier: *boundary bridging, boundary making* and *boundary maintenance*. HB took field-notes during observed lessons. These contained observations and notes on spontaneous questioning of participants to clarify the observed behavior. Subsequent to each observed lesson HB conducted a brief conversation with each CT (physically or in Microsoft Teams) in which they reflected on the boundary spanning behavior displayed during the lesson and the researchers initial interpretations thereof. The researcher EB viewed all video recordings and made additional reflective notes. All transcribed, pseudonymized observation recordings, fieldnotes and video-notes were inserted into NVivo (version 12 pro).

### Data analysis

Two authors (HB,EB) separately read, re-read and coded all transcripts using the codes presented in Figure 1, and subsequently discussed these. They presented preliminary results iteratively to all authors, who provided feedback to enhance credibility and consistency [25]. Data saturation occurred after 17 of the 22 analyzed recordings, subsequent iterations yielded no new insights to the refined codes [26]. Data collection, analysis and coding co-occurred, each informing the other using the constant comparative method within and between observations [27]. When immersing ourselves in the data, we identified nuances in our coding scheme that led to an iterative refinement of the observational instrument, for instance by creating subcategories of boundary bridging, and by dividing CTs’ boundary spanning behavior in multimodal indicators: verbal, nonverbal and by use of tools [28]. With further analysis of observations we noticed co-occurrences between different (sub)categories of boundary spanning behavior. Appendix I describes the steps taken during the development of the observational instrument and the publications on which our methodological choices were based. Further information on this can be requested from the authors. Figure 1 represents the final observational instrument which was used to code the transcripts.

### Reflexivity

We adhered to an interpretative paradigm, where both knowledge and meaning are considered acts of interpretation [29]. Accordingly, our interpretations of CTs’ boundary spanning behavior were constructed through observing them in the natural setting, the social context of teaching, whilst interpreting observations using the theoretic constructs of boundary spanning described above. Credibility in the interpretation was established through continuous dialogue between researchers from different professional backgrounds: HB (PhD-fellow and GP trainee) and EB (educational scientist). Additionally the wider research team was consulted frequently: EdG (educational researcher at associate professor level in the GP training school), MK (professor in Education to connect science and professional practice at the Education Center, University Medical Center) RD (GP, professor in General Practice and head of GP specialty training school) and MB (educational researcher).

## Results

In total, 22 lessons were observed. The lesson content covered a variety of topics: reflection on GP practice experiences (n = 7), clinical lessons (n = 6), interprofessional collaboration (n = 4), communication (n = 2) and GP practice organization (n = 3). This generated over 32 hours of video-material. Participating CTs (four female, two male) had an average of 25 years’ experience as a clinician and four years as a teacher. On average, CTs worked as clinician for 22 hours and as teachers for 17 hours per week.

### Observed boundary spanning behavior of the CT

All three categories of boundary spanning behavior, *boundary bridging, boundary making* and *boundary maintenance*, were observed during lessons. Certain lessons yielded more observations of boundary spanning behavior per lesson than others. On average, it appeared that clinical lessons yielded the most observations, whilst communication lessons yielded the least observations per lesson. We did not explore this further given that our unit of analysis was different types of boundary spanning behavior. The indicators ‘verbal’ and ‘use of tools’ were observed in all (sub)categories, the ‘non-verbal’ indicator was observed in boundary bridging. For each boundary spanning behavior, the data are described below, including illustrative examples. Table 1 presents the number of CTs and lessons in which various boundary spanning behaviors were observed. Boundary bridging has five sub-categories.

**Table 1:** Types of observed boundary spanning behavior.


	# CTS	# LESSONS

**Boundary bridging**		

Integrating own clinical experience	**6/6**	**22/22**

Highlighting significant clinical experiences of others	**6/6**	**21/22**

Normalizing or de-normalizing students’ reported clinical experiences	**6/6**	**21/22**

Encouraging students’ sharing of clinical experiences	**6/6**	**16/22**

Encouraging students to apply theory in practice	**6/6**	**19/22**

**Boundary making**	**6/6**	**14/22**

**Boundary maintenance**	**6/6**	**19/22**


### Boundary bridging

In each lesson, boundary bridging behavior was observed. The five subcategories of boundary bridging behavior as distinguished in Table 1 were observed as follows:

1. Integrating own clinical experience to illustrate theory. This was observed for all six CTs in every lesson: CTs used their own clinical experiences to connect the educational- and clinical contexts, both verbally and with use of tools. CTs also executed this behavior during student- or guest-teacher-led lessons. This boundary spanning behavior was mostly seen in lessons concerning clinical practice. Examples included verbalization of statements such as: ‘*Yes, I do encounter this in my [GP-]practice …*’, ‘*I also missed this diagnosis once…*’, (CT5), and ‘*I will give an example, as a GP I …*’ (CT3). In addition to CTs spontaneously sharing experiences, both the students and the psychologists were observed to ask CTs for their practical experiences, for example, ‘*how do you deal with this in your [GP-]practice?*’ (CT6). CTs mostly shared their experiences after they had given the students an opportunity to report their own experience in practice. The CTs clinical experience often added information to the report or conceptualization of the student. CTs who provided less spontaneous input on their own experiences were frequently asked to share experiences. Tools such as books or apps were used as aids for boundary bridging. CTs recommended these as useful or helpful in the care of their own patients. For instance, CT6 stated: ‘*I even sit down with my patients and install this app on their phone. Because […] I believe they won’t end up using it when you only say it to them*’.
2. Highlighting significant clinical experiences of others. This was observed for all six CTs in 21 of 22 lessons: CTs emphasized when information mentioned by students in the classroom was of clinical importance. This was done verbally by short affirmative words (yes, absolutely, etc.), repeating information or complimenting the students’ input, nonverbally by nodding, headshaking or sounds of (dis)approval, and by use of tools, for example a handout made by the CT with core messages around instrument use in clinical practice.
3. Normalizing or de-normalizing students’ reported clinical experiences. This was observed for all six CTs in 21 of 22 lessons: CTs utilized their experiences as a GP and knowledge of norms in clinical practice to normalize or de-normalize students’ experiences. This was observed verbally, nonverbally and with use of tools. It often occurred subsequent to insecurity or doubt expressed by students during lessons. For example, a student expressed feeling exhausted after independently running a large GP practice for a week. CT3 validated this feeling by displaying a shocked expression and stating: ‘*yes, but it is a [GP] practice with 2500 patients. Such practices are normally run by two doctors, so you have done an extraordinary job*!’. CT3 denormalized the experienced situation through nonverbal and verbal responses. Tools were also used as bridging aids in this category, for instance a pessary as practice material in a clinical skills class. During one observation, a student doubtfully showed a maneuver for inserting a pessary he had seen during his clinical work and CT5 normalized this maneuver by showing how this could be useful.
4. Encouraging students’ sharing of clinical experiences. This was observed for all six CTs in 16 of 22 lessons: CT’s invited students to elaborate on their clinical experiences related to the lesson subject. Typical examples included statements such as: ‘*What do you do when [these patients] are in your consultation room?*’ (CT5). When student discussed cases, CTs often invited other students to react to these with their own experience, for example: ‘*How would others solve this in an elegant manner?*’ or ‘*What would you say in this case?*’ (CT3). CT4 made use of a cloud-based collaborative software program in which students could describe their clinical experiences and questions regarding the lesson subject before start of the lesson and used their casuistry in the lesson.
5. Encouraging students to apply theory in practice. This was observed for all six CTs in 19 of 22 lessons: CTs were observed to encourage students to take future action outside the classroom that deepened the connection between the lesson content and practice reality. They verbally encouraged certain approaches students could use in daily practice. They also used tools, for example recommending books, podcasts and websites. CT3 said: ‘*This book has a very good collection [for working with patients with medically unexplained symptoms]. I advise my patients to read it and often hear it really helped them*”. Similarly, CT1 recommended a website that documented the interactions between alternative medication and standard prescription medication. CTs encouraged self-regulated learning relating to approaches or tools which they perceived as useful when treating their patients.

### Boundary making

Boundary making behavior was observed for all six CTs in 14 of 22 lessons. CTs delineated distinctions between clinical practice and various sources of educational information: medical guidelines, educational materials from the university and practical information coming from clinical supervisors or other specialists. To illustrate, we provide an example for each type of source. CT 3 made a distinction between clinical practice and educational material: ‘*This theory on leadership proposes these subdivisions, I however believe that this is outdated, so I will continue with the following …*’ (CT3). CT6 made a distinction between guidelines and clinical practice: ‘*I always palpate the fibular head [when diagnosing ankle injuries]. This is not written in the guidelines, but it is in my system. […] We should look further than the guidelines*’ (CT6). CT5 separated their own clinical practice and information provided by clinical supervisors: ‘*Although not all clinical supervisors believe that you should offer a consultation in these cases but [I believe] for these things you should, it will prevent a lot of trouble*” (CT5), In addition to these verbal communications, we observed the use of tools to aid boundary making. For instance, CT5 created a short handout of key points when treating a particular patient group, because she felt the official one from the guidelines was too extensive.

### Boundary maintenance

Boundary maintenance behavior was observed for all six CT’s and in 19 of the 22 lessons:

CTs temporarily allowed differences to exist between lesson content and clinical practice, in order to benefit the lesson. CT3 stated *‘It might be convenient that I will give some theory first, so that we can use this later on when discussing the case examples*. And, CT6 stated that the focus of the lesson was on the closure of the consult, where in practice a consult entails more than just its closure. Sometimes tools were used to aid the execution of boundary maintenance: CT5 showed a list with medical topics that could be presented in the upcoming lessons, giving the students a choice on what topics would be more or less useful for their current practice.

Boundary maintenance was often applied in situations where there was shortage of time or a discussion of topics that did not align well with content of lesson and CTs often offered a safety net. They offered that the unaddressed topic could be discussed after lessons or another time (when it better aligned with the content of the lesson or when there would be more time).

### Co-occurrences between different types of boundary spanning behavior

Although all types of behavior were observed independently, the analysis showed that there were notable patterns in the sequence of certain behaviors, these are described below.

When CTs shared their own experience, it was often followed by encouraging students to apply theory in practice. Whilst discussing the placement of spiral contraception CT5 said. “*I made the mistake once to not do the internal exam together with a student and the procedure was complicated by a uterus perforation* (integrating own clinical experience*). When you start doing this [procedure], always ask your clinical supervisor to assess the position of the uterus with you* (encouraging students to apply theory in practice).

CTs were often observed to first normalize students’ experiences and then share their own experiences. A student shared her struggle on dealing with unvaccinated GP assistants, CT3 reacted: “*I totally agree with you, that is really complicated* (normalizing students’ reported clinical experiences). *I work in [place X] where many unvaccinated people live and …* (integrating own clinical experience).

CTs were observed to encourage students to apply theory in practice after boundary making. A student mentioned that their clinical supervisor did not always perform speculum examination during a check-up which is in conflict with the guidelines for practice. In reply, the CT shared that they had observed the same and encouraged the students to adhere to clinical guidelines by saying, *‘That is correct, not every clinical supervisor does this, I didn’t learn it from my clinical supervisor either. But you have to!’*.

To summarize, we found that all three forms of boundary spanning behavior were performed in the classroom – *boundary bridging (with five variants), boundary making* and *boundary maintenance*.

## Discussion

This study observed whether and how CTs enacted boundary spanning behavior in the classroom. Our results provided depth to findings reported on boundary spanning behavior previously in self-report data [9,10,11,12] and identified three categories of boundary spanning. Our results show that CTs mostly attempt to create connections between the classroom and the clinical setting (boundary bridging). Additionally and uniquely, this observational study revealed that boundary spanning behavior in the classroom also involves making- and maintaining boundaries, thereby accentuating differences between the two settings.

*Boundary bridging*, the creation of connections between education and clinical practice, was most frequently employed. This aligns with existing discourse on boundaries between education and clinical practice [24,30], which frequently concerns itself with blurring of boundaries.

Most research on the CTs boundary spanning behavior comes from clinical settings, where CTs wear a white coat and perform clinical duties. Our results show that despite the absence of the white coat in the classroom, some boundary bridging- and boundary making behavior performed here are similar to those described in clinical practice as can be seen in Table 2:

Some boundary spanning behavior we observed in the classroom has not yet been studied or documented as occurring in clinical practice, for example, the behavior of validating students’ feelings whilst *(de-)normalizing students’ experiences* or *highlighting significant clinical information*. It is currently unclear whether this behavior is specific to the classroom or could also manifest in a clinical setting.

**Table 2:** Boundary spanning behavior in the clinical- and classroom settings.


CTs BOUNDARY SPANNING IN THE CLINICAL SETTING (WITH REFERENCES FROM LITERATURE)	CTs BOUNDARY SPANNING IN THE CLASSROOM SETTING (WITH RESULTS FROM THIS RESEARCH

Boundary bridging	

CTs incorporating own clinical experiences into their clinical teaching [5]	integrating own clinical experience to illustrate theory

questioning students and filling any knowledge gaps [31,32,33]	encouraging students’ sharing of clinical experiences in the classroom

CTs encouraging students to take actions outside the workplace, such as supplementary readings [32]	encouraging students to apply theory in practice

- not found in literature	(de)normalizing students’ experiences or highlighting significant clinical information

Boundary making	

CTs explaining to their students how certain skills acquired at the institute should be adapted or avoided in certain clinical situations due to unfeasibility or undesirability [34]	accentuating differences in educational institutes and clinical practice

Boundary maintenance	

- not found in literature	CTs temporarily allowed differences to exist between lesson content and clinical practice, in order to benefit the lesson.


In comparing and contrasting our findings with those from other professions, we found that the credibility of a practitioner in the classroom adds to the legitimacy of the knowledge they present in diverse fields such as teacher training [20] and nursing [13]. Boundary work in most fields as also in medical education is aimed at establishing collaboration across boundaries, whereas business studies also report on competitive boundaries [16]. In teacher training, the classroom setting is more akin to the practice setting than in medicine and allows for role-model behavior to be displayed more so than in medical education. In medicine, the practice environment can present time-sensitive, life threatening situations, which are difficult to reproduce or role-model in a classroom. For such situations boundary making strategies are necessary, potentially more so in medicine than in other academic professions.

### Implications for future research

In future research, the developed observation instrument could be used to study CT boundary spanning behavior in clinical practice. Differences and similarities between the clinical and classroom settings could be observed given that we observed activities in this study that have not previously been reported. Furthermore, it would be interesting to study whether targeted faculty development may enhance the enactment of boundary spanning behavior by CTs. It would be worth exploring how our conceptualization of boundary spanning behavior relates to the theoretic tenets of learning through boundary crossing on the part of the student. Boundary crossing literature states boundaries offer learning potential through four learning mechanisms: identification, coordination, reflection and transformation. The CTs boundary making and maintenance behaviors define the educational context in light of the clinical context (and vice versa), thereby delineating how these practices differ. Positive engagement with the differences prompts students to engage in renewed sense-making process concerning these practices potentially leading to learning through the mechanisms of boundary crossing [24].

Further research might focus on the role of CTs boundary spanning behavior on the professional identity formation of students. This potential resides in the CT’s capacity to act as professional representatives of their field, engaging with students by sharing personal experiences and reflections as GPs. CTs thereby provide students access to the culture, traditions and practices of the GP’s professional community [23], which facilitates a process of professional socialization for students and orientation in the professional community field of GPs [35,36].

### Implications for medical-education institutes

The boundary spanning behavior identified in this study emphasizes the capacity that CTs have to harness boundary spanning to enrich students’ learning. Lesson designs could incorporate methods that illuminate the CTs’ expertise, provide students with opportunities to share practical experiences and stimulate meaningful interactions around the CTs’ expertise and student experiences in comparison or contrast with theory. In teacher training, medical-education institutes could provide support for CTs to effectively navigate their role as boundary spanner within the classroom using boundary bridging and the lesser known boundary making and maintenance strategies. Especially the boundary making and maintenance may enhance students’ reflection on- and learning from the differences and discontinuities between the two setting. It may trigger ‘boundary crossing’ learning mechanism for the students: in particular reflection and identification, but possibly also coordination and transformation. CTs could be educated on the learning potential of positive engagement with boundaries, and receive training on didactic methods they can employ to leverage this potential.

### Strengths and limitations

The use of observation as a data collection method offered valuable insights into CTs’ boundary spanning behaviors in the classroom that was unlikely captured using self-report. The extensive video footage provided a rich source of data, allowing for in-depth analysis and a comprehensive understanding of CTs’ boundary spanning activities. By recruiting CTs from different institutes and recording multiple different lesson subjects, enhanced the diversity and representation within the sample. The use of the constant comparative method during analysis led to a more comprehensive observation schedule, which contained categories not reported in previous publications on boundary work. This observation schedule holds potential as an instrument suitable for conducting comparable observations, including in a clinical setting.

However, we acknowledge the inherent limitations of observational studies, including observer bias. Also, the online lessons may have introduced differences in the dynamics of CT behavior and interaction.

## Conclusion

CTs demonstrate three types of boundary spanning behavior in the classroom – *boundary bridging, boundary making* and *boundary maintenance*. Most boundary spanning activities we observed aimed to bridge the boundaries between education and clinical practice. There were also boundary spanning activities that temporarily accepted or even accentuated differences between the settings, for the sake of student learning. By observing how CTs enact boundary spanning behavior within the classroom setting, this study contributes to an understanding of how CTs use their experience as clinicians in classroom teaching. These insights may contribute to reflection on one’s boundary spanning behavior and to faculty development fostering this type of behavior. The comprehensive observation schedule created within this study holds potential as an instrument suitable for conducting comparable observations.

## Additional File

The additional file for this article can be found as follows:

10.5334/pme.1751.s1 Appendix I. Developmental phases of the observational instrument.
