# Supplementary material for: Boundary Spanning Behavior of Clinician-Teachers in the Classroom. An Observation Study
Source: Perspect Med Educ. 2025 Aug 6;14(1):462–72. doi: 10.5334/pme.1751 (PMC12330804; doi:10.5334/pme.1751)
Supplement: Appendix I. — Developmental phases of the observational instrument. [file pme-14-1-1751-s1.pdf]

Appendix I – Developmental phases of the observational instrument

In the main document, the focus is on the results of observing boundary spanning behavior. To help the reader understand our observational instrument, we provide here a description on the development of the observational instrument to strengthen the transparency of our approach and the trustworthiness of our findings.

The development consisted of the following steps. First, a qualitative protocol and concept instrument was developed based on boundary spanning literature. Second, pilot observations were done to evaluate the usability of the instrument using three lessons and third, continuous refinement of the instrument took place by peer-debriefings after every observed lesson.

Scoping the literature
Given our aim to observe boundary spanning behavior of the CT and lack of publications in this field, we scoped the literature for possible classifications of boundary spanning actions, making our observations more focused. We considered a number of classifications as potentially relevant when searching literature in social [1], organizational [2-5], educational [6], medical [7] and legal [8] science. All classifications use different terms to both describe actions that *lower* the boundaries (with the aim of better collaboration between both settings) and actions that *emphasize differences* between both settings (with the aim to 'separate us from them', distinguish themselves and thereby creating legitimacy). Some also classify actions with the aim to maintain boundaries as they are [1-5], in which Langley et.al. describe this with the aim of - at least temporarily - keeping certain activities in the different settings apart. All three actions we deemed applicable to our observation. Some also described actions that reject boundaries by pretending they do not exist [1,6]. We assumed this rejection would not be applicable to our research.
Finally we conceptualized three forms of boundary spanning actions that according to us could relate to students' learning potential: *boundary bridging*, *boundary making* and *boundary maintenance*.

Pilot observations
HB and EB used the concept observational instrument during three pilot-observations of one CT (CT3). Lesson subjects included intervision, medical theory and organizational theory. When interpreting a CT's behavior as a boundary spanning action, the interpretation of the behavior was written down in the related category. The following main themes were discussed after the pilot observations:

- Boundary making could not be observed in these observations. Based on the literature and our personal beliefs we expected CT's, from their self-oriented dual-role perspective, to explicitly distinguish certain theoretical aspects from practice reality during their lesson. Therefore, the category remained in the instrument for upcoming observations to further examine its observability.
- Boundary maintenance and boundary making were difficult to distinguish from one another. Based on the literature, we decided that boundary making should have a self-oriented perspective and should show a CT emphasizing and allowing differences

to exist between both world. In case of boundary maintenance, we agreed the CT only temporarily allowed differences to exist from a lesson oriented perspective.

- There were two types of indicators of boundary spanning activities observed, of which most were verbal, However also some objects were used by the CT to link both settings were noted.
- Boundary bridging manifested in more diverse variations than initially expected and subcategorizations of the data had added value for understanding boundary bridging behavior. Subcategories were 'integrating own clinical experience to illustrate theory' and 'encouraging students' sharing of clinical experiences. Additionally, 'encouraging students to apply theory in practice' was added as a subcategory, since we observed that the CTs provided the students with options to bridge between the educational- and clinical context themselves.

Further refinement of the instrument

HB and EB used the adjusted instrument separately during the other observations. After each observation, findings were discussed and the usability of the instrument evaluated. Through these peer-debriefing sessions the instrument was further specified and changes to the instrument were made in the following:

- Extra subcategories in boundary bridging were observed: 'normalizing students' reported clinical experiences' and 'highlighting significant clinical experiences of others'.
- It was observed that non-verbal cues also seemed of importance, where the CT used nods or facial impressions to accentuate important of (ab)normal situations brought forward by others. Therefore, non-verbal as an extra indicator was added in the instrument.

References

1. Lamont M, Molnár V. The study of boundaries in the social sciences. Annu Rev Sociol. 2002;28:167-95 DOI: 10.1146/annurev.soc.28.110601.141107.
2. Langley A, Lindberg K, Mørk BE, et al. Boundary work among groups, occupations, and organizations: From cartography to process. Acad Manag Ann. 2019;13(2):704-36 DOI: 10.5465/annals.2017.0089.
3. Zietsma C, Lawrence TB. Institutional Work in the Transformation of an Organizational Field: The Interplay of Boundary Work and Practice Work. Adm Sci Q. 2010;55(2):189-221 DOI: 10.2189/asqu.2010.55.2.189.
4. Liljegren A. Pragmatic professionalism: micro-level discourse in social work. Eur J Soc Work. 2012;15(3):295-312 DOI: 10.1080/13691457.2010.543888.
5. Abbott A. The system of professions: An essay on the division of expert labor. Chicago, IL, US: The University of Chicago Press; 1988. xvi, 435-xvi, p.
6. Risan M. Negotiating professional expertise: Hybrid educators' boundary work in the context of higher education-based teacher education. Teach Teach Educ. 2022;109:103559 DOI: https://doi.org/10.1016/j.tate.2021.103559.
7. Schölvinck A-FM, Pittens CACM, Broerse JEW. Patient involvement in agenda-setting processes in health research policy: A boundary work perspective. Sci Public Policy. 2020;47(2):246-55 DOI: 10.1093/scipol/scaa001.

8. Liu S. Boundary Work and Exchange: The Formation of a Professional Service Market. Symb Interact. 2015;38(1):1-21 DOI: https://doi.org/10.1002/symb.137.
